# Bullous pemphigoid as an injection site reaction of glatiramer acetate

**Published:** 2019-10-07

**Authors:** Seyed Mohammad Baghbanian, Maryam Ghasemi, Somayeh Sheidaei, Zohreh Hajheydari

**Affiliations:** 1 Department of Neurology, School of Medicine, Mazandaran University of Medical Sciences, Sari, Iran; 2 Department of Pathology, School of Medicine, Mazandaran University of Medical Sciences, Sari, Iran; 3 Immunogenetic Research Center, Mazandaran University of Medical Sciences, Sari, Iran; 4 Department of Dermatology, School of Medicine, Mazandaran University of Medical Sciences, Sari, Iran; 5 Invasive Fungi Research Center, Bualicina Hospital, Mazandaran University of Medical Sciences, Sari, Iran

**Keywords:** Bullous Pemphigoid, Glatiramer Acetate, Multiple Sclerosis

Glatiramer acetate (GA) is one of the well-tolerated disease-modifying therapeutic options, which is commonly administered subcutaneously in patients with multiple sclerosis (MS). The current study aimed at defining a bullous pemphigoid (BP) skin reaction in a patient with MS receiving treatment with GA.

A 29-year-old women with MS, receiving GA treatment within the past nine months, November 11, 2018, was admitted to our MS clinic due to itching skin eruptions at the site of injection. Her disease was started at February 12, 2017 with left optic neuritis; and because of six periventricular and eleven juxtacortical brain magnetic resonance imaging (MRI) lesions without any enhancement, lumbar puncture was done for her. Due to positive cerebrospinal fluid (CSF) oligoclonal band, MS was diagnosed for her. At that time, she refused to start disease modifying treatment. GA was started 9 months before admission for her. 

After dermatologic consultation, the dermatologist defined the lesions as fluid-filled and blistering at the site of injection without mucosal involvement (Figure 1).

**Figure 1:**
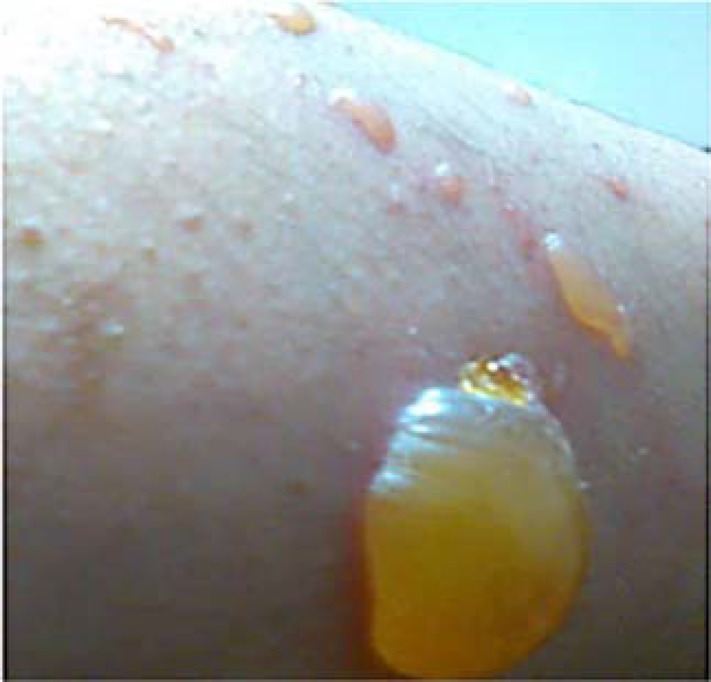
Large, fluid-filled blisters

***Timeline:*** Skin lesions appeared exactly at the GA injection site.

***Diagnostic Assessment:*** The histological examination revealed hyperkeratotic and acanthotic epidermis with subepidermal blister formation that contained fibrin deposition, eosinophils, and neutrophils. Edematous papillary dermis showed congested blood vessels with mixed perivascular inflammatory cells infiltration (Figure 2). 

**Figure 2:**
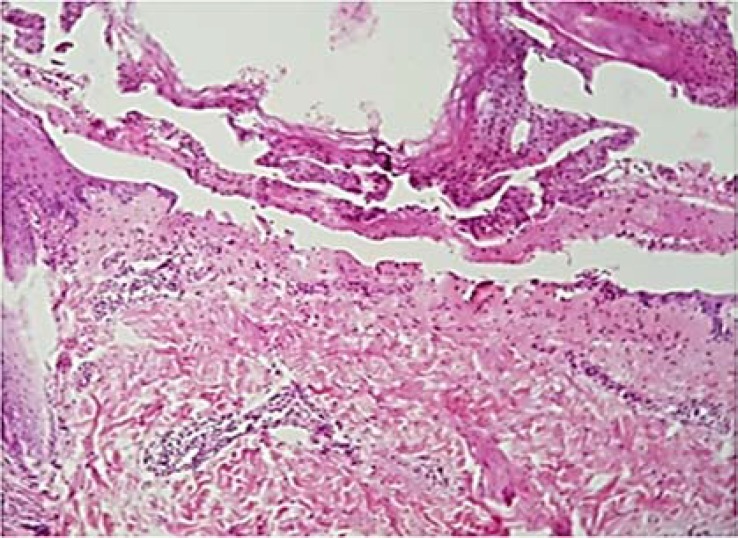
The hematoxylin and eosin (H&E) staining shows subepidermal blister (magnification: 100×).

Direct immunofluorescence showed continuous linear IgG and partial C3 deposition in basement membrane zone. The immunoreactivity with anti-IgA and anti-IgM was negative (Figure 3), and BP was diagnosed in the patient. 

**Figure 3:**
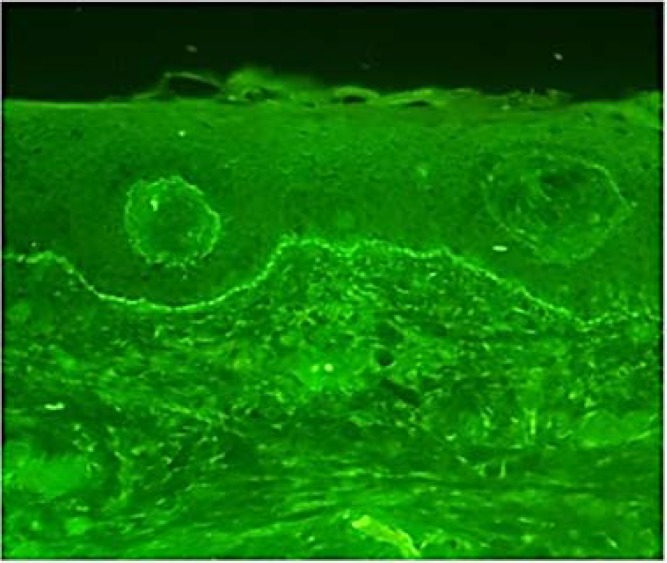
Direct immunofluorescence shows continuous linear immunoglobulin G (IgG) deposition in basement membrane zone.

***Therapeutic intervention:*** One month after GA discontinuation, the skin lesions resolved completely. 

***Follow-up and Outcomes:*** No scarring appeared at the lesion site after recovery.

BP is generally described as an immune-mediated skin disorder. Autoimmunity against BP antigens, Ag1 and BPAg2, in the lower layer of epidermal keratinocytes characterizes the pathogenesis of BP.^1^ The incidence of BP is reported to be 14 to 43 cases per million populations in Europe.^2^ BP is associated with neurological disorders such as MS,^1^ Parkinson’s disease, and dementia, and cardiovascular disease.^1^

A cross-reaction between autoimmunity against BPAg1 and neurological disorders is hypothesized. The frequency of MS increases in patients with BP both during and after diagnosis.^1^ On the other hand, the risk of MS in patients with BP is reported to be six times higher than that of the matched general population.^1^ BP mainly affects elderly patients, and in the first reports of BP comorbid with MS, the mean age at skin reaction onset was reported to be 49 and 62 years;^3^^,^^4^ however, in the current study, skin reactions were detected only nine months after MS diagnosis at the age of 29 years. 

In previous reports,^3^^,^^4^ no association was identified between skin eruptions and site of injection. Another case report of MS and BP indicated skin eruptions at the site of indwelling catheter of bedridden patients.^5^ It seems that BP is mostly comorbid with MS, compared to other autoimmune disorders. Changes in the earlier stages of the disease and the relationship between its pathogenesis and clinical course remain unknown. Nonetheless, since BP mortality increases by time,^1^ the prognosis of MS may be affected. To the best of our knowledge, this is the first report of BP as an injection site reaction of GA. Therefore, the patients with MS should be asked about any injection site reactions.

## Notes:

**How to cite this article:** Baghbanian SM, Ghasemi M, Sheidaei S, Hajheydari Z. Bullous pemphigoid as an injection site reaction of glatiramer acetate. Iran J Neurol 2019; 18(4): 187-9.
